# A meta-analytic perspective on the valid use of subjective human judgement to make medical school admission decisions

**DOI:** 10.1080/10872981.2018.1522225

**Published:** 2018-10-05

**Authors:** Clare Kreiter, Marie O’Shea, Catherine Bruen, Paul Murphy, Teresa Pawlikowska

**Affiliations:** a Department of Family Medicine, Office of Consultation and Research in Medical Education, University of Iowa Carver College of Medicine, Iowa City, IA, USA; b Health Professions Education Center, Royal College of Surgeons, Dublin, Ireland; c Technology Enhanced Learning Manager, Royal College of Surgeons, Dublin, Ireland; d Information Specialist, Royal College of Surgeons, Dublin, Ireland; e Director of Health Professions Education Centre, Royal College of Surgeons, Dublin, Ireland

**Keywords:** Admissions, medical education, meta-analysis, admission interviews, holistic review

## Abstract

While medical educators appear to believe that admission to the medical school should be governed, at least in part, by human judgement, there has been no systematic presentation of evidence suggesting it improves selection. From a fair testing perspective, legal, ethical, and psychometric considerations, all dictate that the scientific evidence regarding human judgement in selection should be given consideration. To investigate the validity of using human judgements in admissions, multi-disciplinary meta-analytic research evidence from the wider literature is combined with studies from within medical education to provide evidence regarding the fairness and validity of using interviews and holistic review in medical school admissions. Fourteen studies, 6 of which are meta-analytic studies that summarized 292 individual studies, were included in the final review. Within these studies, a total of 33 studies evaluated the reliability of the traditional interview. These studies reveal that the interview has low to moderate reliability (~.42) which significantly limits its validity. This is confirmed by over 100 studies examining interview validity which collectively show interview scores to be moderately correlated with important outcome variables (corrected value ~.29). Meta-analyses of over 150 studies demonstrate that mechanical/formula-based selection decisions produce better results than decisions made with holistic/clinical methods (human judgement). Three conclusions regarding the use of interviews and holistic review are provided by these meta-analyses. First, it is clear that the traditional interview has low reliability and that this significantly limits its validity. Second, the reliable variance from interview scores appears moderately predictive of outcomes that are relevant to consider in medical school admission. And third, the use of holistic review as a method of incorporating human judgement is not a valid alternative to mechanical**/**statistical approaches as the evidence clearly indicates that mechanistic methods are more predictive, reliable, cost efficient, and transparent.

## Introduction

The most recent survey of admission practices at North American medical schools suggests that admission programmes make substantial use of subjective judgement/assessment [1]. Despite the introduction of more objective approaches such as situational judgement assessments, multi-mini interviews, and other high-structure interview-like techniques, scores from the traditional subjective interview continue to assume a prominent role. In addition, holistic committee review as a means of incorporating subjective human judgement is being increasingly promoted and employed. Popular support for the use of subjective assessment is conveyed in the many published perspectives expressing a need for an individualized evaluation of each medical school applicant [2–4]. Despite the widespread use of, and support for, subjective human judgement/assessment, there is no evidence-based consensus on how it can best be employed in medical school admission. While it is well-known that interviews and subjective holistic review of an applicant plays an influential role, how these subjective judgements impact the integrity of the admission decision is not well understood. Given that selection is governed to a significant degree by the subjective assessment of an applicant, it seems prudent to ask how this impacts the validity of our admission decisions. To answer this question, a review and summary of the research literature is an important first step. As this cross-disciplinary research synthesis will show, the existing evidence provides generalizable and reasonably conclusive findings that provide substantial insight into how subjective assessment affects the fairness, reliability, and validity of the decisions that determine who will be allowed to become a physician.

To select their students, medical colleges typically rely on their faculty and staff to perform two subjective assessments. First they rate an applicant’s performance on an admission interview, and second, an admissions committee subjectively combines the information in the applicant’s file to generate a rating, ranking, or decision that ultimately determines whether an applicant is accepted or rejected. In addition, these two subjective procedures potentially reinforce each other as holistic review favors a subjective weighing of interview scores over the application of evidence-based weights [5]. Survey research documents the influential roles of the traditional interview and holistic committee review in medical school admission [1,6–9].

Interviews and holistic review typically requires thousands of human-resource hours from a medical school’s most highly paid faculty and staff, and are the primary ways in which subjective judgements are utilized in medical school admission. Given the cost, ubiquity, and consequence of using interviews and holistic review, it would be instructive to ask admission specialists who rely on these techniques whether they could provide an evidence-based rationale for their use. The answer would almost certainly be ‘no’, as the medical education literature does not currently provide an evidence-based validity argument to support their use. While this draws attention to the fact that many medical colleges may not be optimally managing their human capital, it does not necessarily imply that subjective judgements should be removed from the decision process. Rather, it indicates a need to systematically review and interpret the research in a way that will allow a valid utilization of the subjective assessments that are performed by the faculty and staff of the medical college.

While medical educators appear to believe that medical school admission should be governed, at least in part, by human judgement, and our admission practices indicate that we value these judgements, there has been no systematic presentation of evidence that suggests it improves selection. For proof, scientific research is required. While scientific evidence can challenge our intuition and disrupt established practice, there is a general understanding that building on testable evidence is the best way to achieve progress. Although scientifically inspired change can be slow, modern medicine’s subscription to the scientific method and evidence-based practice suggests that research will ultimately be a positive change agent for medical education [10].

This leads to the obvious question of why medical colleges use techniques that are not supported by research. Some have argued that our national organization is responsible as their advocacy for subjective assessment is not supported with scientific validity evidence. This cannot be the sole reason however, as both the traditional interview and holistic committee review were in widespread use long before any organizational efforts to promote them [1,8,11,12]. Other likely explanations are that interviews and holistic committee review reflect our preference for tradition, unfamiliarity with validity concepts, the physician educator’s attachment to clinical/intuitive approaches, and more recently, attempts to avoid legally prohibited quota-based methods for attaining diversity.

While medical educators may believe human judgement is required to make admission decisions, they also intuitively understand that it is inappropriate to base high-stakes admission decisions on techniques that ignore or inefficiently utilize valid indicators of a prospective medical student’s ability to excel in the profession. To utilize human judgement in a way that preserves the predictive value of the measures employed, it is critically important that medical educators understand the formal requirements for the fair and valid use of assessment in high-stakes selection. It is especially important to realize that it is not sufficient to simply collect reliable and valid indicators of an applicant’s potential; it is necessary that the obtained measures be used in a fashion that is reliable and valid as well [13–15]. Useful information should not be discarded during the selection process, and demonstrating the logic of how assessment information is utilized is a critical step in establishing the validity of the selection methods and measures employed.

Currently, our national organization promotes holistic review as a technique for improving upon the quality of admission decisions that could alternately have been made with more transparent and objective algorithmic**/**mechanical approaches. With what appears to be an implicit assumption that subjective assessments do not require objective quantitative validity evidence, the *Association of American Medical College*s (*AAMC*) promotes subjective holistic review with subjective qualitative studies and testimonials [5]. It is important however to consider this approach from a validity perspective and ask whether relying solely on qualitative methods is consistent with the validity standards for high-stakes selection. From a fair testing perspective; legal, ethical, and psychometric considerations each dictate that all evidence, both qualitative and quantitative, regardless of whether it is likely to support or refute a proposed interpretation, must be given consideration [13–15]. Yet, despite the AAMC’s own recommendation that research should ‘identify what in the holistic review admission process is working and what is not,’ the *AAMC* has not presented quantitative evidence of prediction or reliability to support their recommendation for the use of holistic review in medical school admission [16]. While some have pointed out a need for such evidence, empirical investigations have not been conducted, and meta-analytic summaries of the existing validity research reflecting on the use of interviews and holistic review in medical school admissions has not been considered [11,12,17].

This review is an initial step in providing the quantitative evidence needed to generate a logical/scientific validity argument for the use of subjective assessment in medical school admission. It summarizes the relevant literature by combining studies from within medical education with the multi-disciplinary meta-analytic research that has been conducted on the two most commonly employed subjective assessments (interviews and holistic committee review) used in medical school admission. Holistic review uses human judgement to combine information about an applicant to make a decision. Traditional interviews employ human judgement to rate an applicant based on interview performance and sometimes other information as well. This review evaluates the validity evidence for these two most commonly used methods of employing subjective human judgement in admission. It examines three aspects related to validity. First it evaluates the reliability of the traditional interview. Second, evidence for the predictive validity of the traditional interview is presented. And third, it evaluates whether using holistic decisions as a way to introduce human judgement improves decisions compared to using statistical/mechanical approaches. This evidence-based review provides the information needed to construct a valid approach to using subjective judgements to make medical school admission decisions. The evidence will help promote the development of an evidence-based approach for utilizing subjective assessments in a fashion that will maximize the fairness, validity, and reliability of selection decisions.

## Materials and methods

To generate an evidence-based meta-analytic perspective on the fair use of subjective methods in medical school admission, research from medical education, health science education, medicine, psychology, personnel/employment, education, and the wider social sciences is considered. This critical review brings together a wide range of studies that provide multi-disciplinary quantitative evidence characterizing the measurement properties of interviews and holistic review. Searches were limited to peer-reviewed scholarly sources: research papers and meta-analytic reviews. As far as interviews, we limited inclusion in the study to traditional unstructured and semi-structured interview methods that relied primarily on human judgement, and therefore excluded research on more objective structured clinical examination (OSCE)-based techniques such as the multi-mini interview (MMI). To answer the measurement and validity-related questions, existing meta-analytic studies from the wider literature are combined with individual medical education studies to provide the validity evidence needed to guide the fair utilization of interviews and holistic review in medical school admission [18,19].

### Identifying relevant studies

Comprehensive literature searches were carried out in May 2017 in the following databases: MEDLINE, EMBASE, PsychInfo, Web of Science, ERIC, CINAHL, Cochrane, and Health Business Elite. The search strategy was designed with the assistance of an expert Medical Education librarian and was constructed with the following criteria in mind: [Admission to programme] AND [Student selection processes] AND [Methods/Means] AND [Setting/Population]. Initially the search was piloted in MEDLINE and then tailored to all final databases. The search was slightly adapted for the Health Business Elite database [admission interview selection]. Search terms were modified as relevant per database to take into consideration British and American word variants. The references of the included articles were examined to identify additional relevant studies.

### Study selection and synthesis of results

Search results were imported into Endnote (version X8.0.2 for Windows®). Inclusion criteria were published peer-reviewed research papers and reviews which contained numerical data such as reliability coefficients, G coefficients, validity coefficients, or quantitative statistical comparisons. We excluded editorials, opinion pieces, dissertations, theses, books, and non-peer reviewed articles. Studies reported in languages other than English were excluded. No restrictions were placed on year of publication.

**Figure 1.**
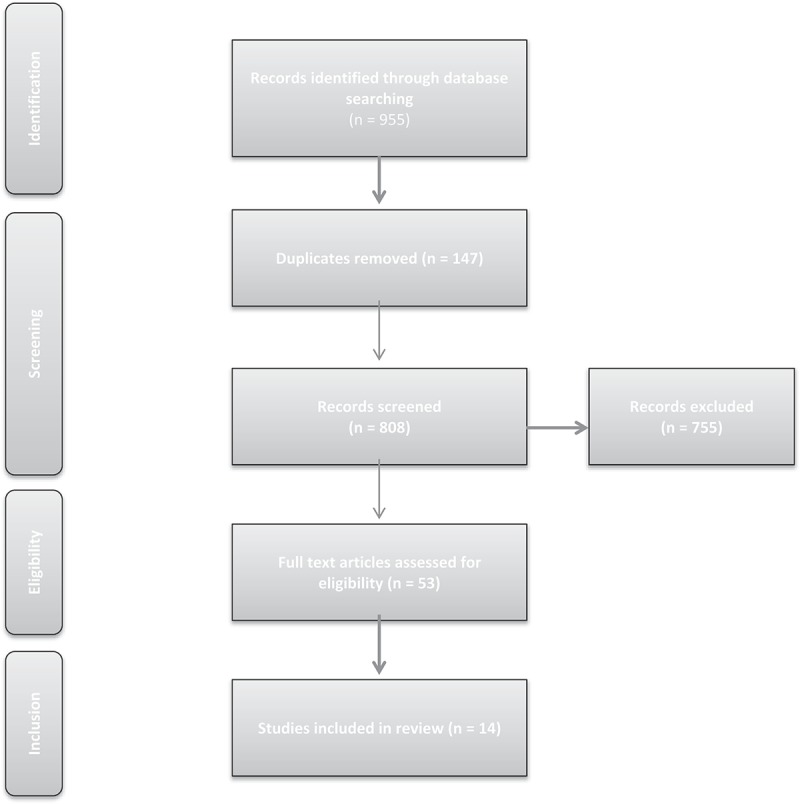
Data search methods.

First-round screening consisted of independent review of abstracts and titles by two members of the research team (MTOS and CB). Full papers were sourced for second round screening if both reviewers considered the paper potentially relevant. Any disagreements were resolved by a third reviewer (CK). Two reviewers (CK and TP) then assessed the eligibility of full research papers selected for according to the above criteria. See Figure 1 for PRISMA diagram.

The searches identified 955 references, de-duplication reduced them to 808. Seven hundred and fifty-five articles were excluded in first-round screening, with 53 full text articles assessed for eligibility, three of which were included following reference searching. Research studies that had been already incorporated into existing meta-analytic were not used individually in this study.

## Results

The study selection process outlined in Figure 1, produced 14 articles for the final review: 11 from the USA, 2 from Canada, and 1 from Bahrain.

### The selection interview

#### The reliability of interviews

While there are studies investigating the reliability and validity of medical school admission interviews, there exist a far more extensive literature examining the interview in other related selection contexts. To benefit from this wider research, this review considers studies on traditional selection interviews conducted across health science education and employment. While there are meaningful differences between selection for employment and selection for medical education, in many countries, nearly all applicants who accept admission to medical school do go on to graduate and become employed as physicians. This means medical school admission is effectively functioning as a form of employment selection [20–22]. With the near universal matriculation from medical education to practice, the validity argument for medical school admission must consider the ability to predict physician performance [22]. This is widely acknowledged in medical college mission statements which invariably reflect the inextricable link between academic and professional achievement [23].

Interpreting the literature on interview reliability is complicated by the fact that there are a number of ways in which error influences an interview score. The best estimate of reliability is a coefficient incorporating all sources of random errors, allowing an inference regarding the interview’s upper limit on validity. In other words, an optimally informative estimate of reliability will convey how consistently an interview score can be reproduced upon a complete replication of the interview. This can best be estimated as the correlation between performances for a group of applicants who repeat the entire interview process [24]. That is, the correlation between two interviews of the same applicants on different occasions using different raters and questions. This correlation will represent how consistently applicants can be ranked and will include the most important and influential sources of random error. In medical education, where generalizability (G) theory is the preferred approach to estimating reliability, these sources of error are labelled: *rater, question*, and *occasion*. The research tradition in the employment and personnel management sciences uses a more classical approach to estimate each source of error with research designs that isolate error sources within the interview. This approach employs inter-rater or intra-class correlation coefficients (ICCs) and coefficient alpha for varied interview formats to provide estimates of the *conspect, random response*, and *transient* error that attenuates the correlation between complete replications of an interview [24,25]. Although inconsistent terminology is a source of confusion, the two sets of labels describe essentially the same sources of error. In addition, there is consensus across disciplines that the most meaningful and informative coefficient of reliability will provide an estimate of score stability across complete replications of an interview. Studies that report coefficients reflecting only limited sources of measurement error do not provide an estimate of score consistency upon repeating the entire interview process. For example, an ICC estimating the correlation between two interviewers present at the same interview, or an alpha internal reliability coefficient calculated across questions within a single interview, will each estimate just one source of error; rater and question-nested-within occasion, respectively. Because these estimates of reliability do not include a comprehensive estimate of error, they do not permit an inference regarding the maximum attainable validity and are not included in this research review.

Table 1 summarizes findings from 5 of the 14 final papers. These 5 papers include a total of 33 studies of interview reliability and provide an estimate of the average reproducibility of unstructured and semi-structured interviews across 2325 applicants. The first entry in the table summarizes a meta-analysis of reliabilities from the employment literature [24]. The remaining entries in the table document four studies from medical education; three using G study methodologies and one using coefficient alpha calculated on total scores across complete replications of the interview [26–29]. Reported in the second to last row of Table 1 is the simple average (weighted by the number of studies) reliability (*r*_a_ = .42). Displayed in the last row of the table is the theoretical maximum possible correlation with a perfectly reliable criterion (the upper limit on the attainable validity) that is calculated as the square root of reliability and is equal to *r*_x__ʹ__y__ʹ_ = .65 [30,31]. The next section of this review will evaluate the degree to which the maximum attainable validity is achieved in practice by examining the interview’s correlations with the criterion variables reported in the literature.

**Table 1.** Estimates of traditional (unstructured/semi-structured) selection interview reliability.

Study [reference no.]	Application	Type of study (*N* of studies) {*N* of applicants}	Reliability
Huffcutt [24]	Personnel selection/employment	Meta-analysis (28) {1584}	*r* = .44
Kreiter et al. [26]	Medical school admissions	G study (1) {92}	*r* = .32
Axelson et al. [27]	Medical school admissions	G study (1) {168}	*r* = .31
Shaw et al. [28]	Medical school admissions	Repeat Interviews – alpha coeff. (2) {471}	*r* = .23
Hanson et al [29]	Medical school spec program admission	G study (1) {10}	*r* = .46
Weighted average		(33) {2325}	*r*_a_ = .42
Upper limit on validity			*r*_x__ʹ__y__ʹ_ = .65

#### The validity of interviews

The literature used to establish validity relies on the interview’s ability to predict various measures of academic, clinical, and/or employment success. To estimate the true validity of the interview, it is necessary to correct the observed correlations for the unreliability of criterion measures and for the range restriction that results from selection. Corrected correlations are more accurate in characterizing the true validity because the observed (uncorrected) correlation between the interview and the criterion will depend upon how accurately (reliably) researchers are able to measure the criterion variable (the outcome) and the degree to which the score range of both the predictor and criterion are restricted by selection. In other words, observed correlations will underestimate the true validity of a predictor when outcomes are measured imprecisely and when the range of values observed is restricted by selection of the highest values. Both these influences attenuate (reduce) the observed correlation. This meta-analytic summary reports both observed correlations and corrected correlations using the single (rather than the double) correction for attenuation due to reliability [32]. While the disattenuation for low reliability made a substantial impact on the corrected estimates, range restriction played a relatively smaller role due to the low reliabilities suppressing correlations and producing minor restrictions of range on the outcome variables. When studies did not report corrected values, they were estimated with coefficients from similar studies and entered into the table.

Table 2 provides a listing of 106 published validity outcomes as identified in 7 of the final 14 papers included in this review. The first two entries in the table list meta-analytic outcomes from across the academic healthcare literature [33]. Goho et al. report the results of two meta-analytic studies using effect sizes that are converted to correlations for display in Table 2 [33]. The first study summarizes academic performance, and the second study summarizes clinical performance during the students’ education. Corrections for range restriction and criterion unreliability were generated using estimates from similar studies in the literature. The third and fourth entries in the table report comprehensive meta-analyses for unstructured and semi-structured interviews from the employment literature, and both performed corrections for criterion unreliability and range restriction [34,35]. The fifth entry in Table 2 reports on validity estimates from medical education as correlations between interviews and communication scores on a licensure test and between interviews and the academic achievement component of a licensure test [36]. The sixth entry by Al-Nasir et al. documents the correlation between interviews and first-year medical school grades [37]. The seventh entry in the table reports on the correlation between the interview and performance during clinical training [38]. The eighth entry displays the correlation between the interview and ratings of clerkship performance and a licensure test [39]. The average uncorrected coefficient (weighted by number of coefficients) was *r*_a_ = .15. The average corrected coefficient across studies was *r*_ac_ = .29. The results displayed in Table 2 tended to show higher correlations for non-academic outcomes. This may be an important consideration for the use of the interview given the priority and recent emphasis on developing predictors of non-academic performance outcomes [40]. Generally, these results show that the reliable variance of the interview, while far from attaining the maximum attainable prediction of any outcome variable (*r*_x__ʹ__y__ʹ_ = .65), does appear moderately associated with outcomes that are relevant in medical school admission.

**Table 2.** The validity of traditional (unstructured/semi-structured) selection interviews for predicting academic, clinical, and employment outcomes.

Study [reference no.]	Application	Type of study (no. of coefficients) {no. of applicants}	Observed correlation	Corrected validity coefficients
Goho et al. [33]	Academic health care – post-secondary admission	Meta-analysis – academic perform. (19) {4488}	*r* = .03	*r*_c_ = .05
Goho et al. [33]	Academic health care – post-secondary admission	Meta-analysis – clinical perform. (10) {1283}	*r* = .08	*r*_c_ = .15
McDaniel et al. [34]	Employment	Meta-analysis – job performance. (39) {9330}	*r* = .18	*r*_c_ = .33
Le & Schmidt year [35]	Employment	Meta-analysis – job performance (34) {8,985}	*r* = .18	*r*_c_ = .41
Kulatunga, et al. [36]	Medical school admissions	LMCC I & II communication/clinical-academic (1) {97}	*r* = .24/ *r* = .08	*r*_c_ = .30/ *r*_c_ = .09
Al-Nasir et al. [37]	Medical school admissions	First-year grade (1) {68}	*r* = .28	*r*_c_ = .32
Murden et al. [38]	Medical school admission	Clerkship perform. (1) {435}	*r* = .22	*r*_c_ = .28
Meredith et al. [39]	Medical school admission	Clerkship perform/licensure (1) {85}	*r* = .32/ *r* = .08	*r*_c_ = .38/ *r*_c_ = .12
Average (a)		(106) {24,771}	*r*_a_ = .15	*r*_ac_ = .29

### Holistic review

When considering the utility of holistic review, it is important to point out that this technique represents one of two general methods that are used to combine information for making a decision. The two methods are the *mechanical prediction* method and the *clinical judgement* method. *Mechanical prediction* uses equations based on statistical models (e.g., multiple regression, discriminate analysis, unit weighted sums) to maximize the prediction of either criterion outcome variables or the previous decisions of experts. Holistic review is a classic example of the *clinical judgement* method which uses raters or judges to subjectively combine data in order to classify or make a decision about an individual or an object. Mechanical methods are 100% reproducible (perfectly reliable), while clinical methods contain rater-related error. Sometimes these two methods disagree. For example, in medical school admission, one method might admit an applicant while the other does not. Instances of disagreement provide an opportunity to study and compare the validity of the two methods.

When employing the clinical method, experts typically view a wide array of information about an object or individual before making a decision. For example, an investment consultant might examine and weigh economic conditions, stock valuations, technical reports, market histories and other data before recommending the purchase of a stock. In a similar fashion, physicians often subjectively weigh and combine multiple sources of information about a patient before making a diagnosis. The holistic review of medical school applicants uses the clinical method to make admission decisions. The holistic review uses human judges or committee members to evaluate and combine applicant information contained in the applicant’s file to make a decision about the applicant. In contrast, the mechanical method statistically weights the codified applicant information. The clinical method and the mechanical method have been compared in dozens of studies investigating the quality of decisions in many different contexts. While academic medical school admission is our primary focus, the strong generalizability of findings comparing the methods across applications suggests a review across contexts will be useful for understanding the clinical method’s (holistic review) application in medical education. Table 3 provides a meta-analytic summary of the research comparing the holistic/clinical method with the mechanistic/formulistic approach.

**Table 3.** Summary of meta-analytic studies of the mean effect size difference between mechanical (algorithmic/formulistic/statistical) vs. clinical (holistic/subjective) decisions.

Study [reference no.]	Application	Sample sizes (No. of Studies) {No. of subjects}	Outcome *(mechanical – clinical) =* effect size difference (ESD) = effect size diff.
Grove et al. [41]	Health-related, diagnosis, prognosis/psychological, behavioural	(136) {15,000+**}**	(.52 – .43) = + .09 ESD = + .09
Kuncel [42]	Job performance	(9) {2548}	(.44 – .28) = + .16 ESD = + .16
Kuncel [42]	Academic selection – GPA	(6) {897}	(.58 – .48) = + .10 ESD = + .10
Kuncel [42]	Work attainment, work training, non-grade acad.	(10) {1334}	(.40 – .33) = + .07 ESD = + .07
**Average across meta-analyses**	Across applications	(161) {~ 20,000}	ESD = + .11

Table 3 summarizes a total of 161 studies as identified in two of the 14 papers included in this review. The first entry in the table describes 136 of those studies comparing behavioral and health-related decisions using mechanical and clinical methods [41]. The mechanical approach tended to produce superior decisions. The second, third, and fourth entries in the table report results from a meta-analytic study in three different contexts [42]. Nine studies across 2548 subjects examined job-related performances and showed mechanical selection to be more effective in selecting high performing employees. Six studies investigating grade point average (GPA) outcomes from over 800 applicants showed mechanical methods to perform better than holistic/clinical methods for selecting applicants to academic programmes. Lastly, five studies examined job promotion and advancement using 683 applicants and showed mechanical prediction to be more efficient. Across applications, the mechanical method outperformed the clinical approach with an average effect size advantage of +.11 SD. Across studies, the mechanical method is shown to provide superior decisions compared to those made with the clinical/holistic approach.

## Conclusions/discussion

This critical review summarizes the research across 300 studies and provides important evidence for designing reliable, valid, and fair selection procedures that incorporate a subjective component. Regarding the use of interviews, it is clear that the traditional interview has low reliability that significantly limits its validity. However, the reliable variance from interview scores does appear moderately predictive of outcomes that are relevant in medical school admission. These results imply that if traditional subjective interviews can be formatted to yield a reliable score (e.g., by engaging an applicant in multiple independent interviews), it might significantly enhance its value as a subjective assessment of an applicant’s potential as a medical student and physician [27,29].

The meta-analysis shows the use of holistic review is not supported by quantitative validity evidence. Given that mechanistic methods are more predictive, transparent, reliable, and economical, holistic review cannot be regarded as a valid alternative to mechanistic or statistical approaches. Because admission programmes are obligated to protect the public trust and ensure fairness, effectiveness and transparency are critical in the high-stakes environment of selection for medical school. From both a fairness and validity perspective, the tendency for holistic review to obscure the decision process and produce inferior outcomes relative to mechanistic or statistical approaches means holistic review cannot be recommended. Since measurement professionals and social scientists can reasonably be expected to be aware of the research reviewed here, and because no positive validity evidence for holistic review in medical education has been provided, recommendations for the use of holistic review in medical school admission should be regarded as contrary to the scientific evidence.

In the light of the research on holistic review and the traditional interview, it is important to address the implications for best practices. For utilizing subjective input within the selection process, it is important to remember that mechanistic selection does not remove all subjective decisions from selection. Formula-based statistical or mechanical methods often contain subjective judgements in quantitative form regarding applicants and class characteristics. The most important difference between holistic and mechanical approaches is that mechanistic methods will use and apply both subjective and objective decision information uniformly across all applicants. While there is often a tendency for mechanical decisions to place a higher value on objective evidence of prediction, this is not always the case.

The weights assigned to applicant attributes and class characteristics within the mechanistic approach are usually a reflection of explicitly stated values that can indeed be quite subjective in nature. For example, decisions regarding the relative importance of communication skills, intellectual ability, and ethnicity/race are inherently subjective because objective evidence regarding the relative importance of each does not exist. While all algorithmic/mechanistic methods can incorporate subjective aspects, the constrained optimization (CO) method is the most explicit and versatile in representing subjective decisions related to class composition and applicant attributes within a mechanistic framework [43–45]. CO methods are preferable to holistic review because they are transparent and treat all applicants equally while providing flexibility for programmes seeking to implement both subjective and objective policy decisions related to both class and applicant characteristics.

The desire to use human judgement in the decision process is understandable since there is evidence that emotional/intuitive pre-conscious choices that might influence an interview rating for example, can in some instances be advantageous. Research on the Iowa Gambling Task clearly shows that individuals can make strategic decisions long before forming any explicit cognitive understanding of why that decision makes sense [46]. While we may have good reason for desiring to use human judgement to select medical students, it is essential that we objectively evaluate which methods work and the consequences of using them. It is important to show that our subjective decisions do not render the selection process less reliable or less valid. The research presented here provides important evidence for applying subjective methods for making medical school admission decisions.
